# Production of nanoparticles from natural hydroxylapatite by laser ablation

**DOI:** 10.1186/1556-276X-6-255

**Published:** 2011-03-25

**Authors:** Mohamed Boutinguiza, Rafael Comesaña, Fernando Lusquiños, Antonio Riveiro, Juan Pou

**Affiliations:** 1 Dpto. Física Aplicada, Universidad de Vigo, Lagoas-Marcosende, 9, Vigo 36310, Spain

## Abstract

Laser ablation of solids in liquids technique has been used to obtain colloidal nanoparticles from biological hydroxylapatite using pulsed as well as a continuous wave (CW) laser. Transmission electron microscopy (TEM) measurements revealed the formation of spherical particles with size distribution ranging from few nanometers to hundred nanometers and irregular submicronic particles. High resolution TEM showed that particles obtained by the use of pulsed laser were crystalline, while those obtained by the use of CW laser were amorphous. The shape and size of particles are consistent with the explosive ejection as formation mechanism.

## Introduction

Nanoparticles represent an important object of investigation in the field of biomaterials due to the new properties and functionalities obtainable when operating at nanoscale [1-3]. Calcium phosphate compounds in particular are getting special attention as biomaterials due their characteristics to induce bone-integration and to anchor rigidly prostheses or implants to the bone [4]. Among them hydroxylapatite (HA), Ca_10_(PO_4_)_6_(OH)_2_, has a special importance because of its similarities with the mineral constituents of bones and teeth, where this material is present as nanometric particles with a platelet shape [5] giving them their physiochemical properties. On the other hand, it has been reported that the use of β-tricalcium phosphate (β-TCP), Ca_3_(PO_4_)_2 _in nanosize scale and low crystallinity improves the bioactivity [6,7].

There are different and diverse techniques for producing calcium phosphate nanoparticles, such as aqueous solutions [8], the templating technique to achieve nano-porous hydroxylapatite structure [9], or the microwave irradiation to synthesize hydroxylapatite nanostructures [10], etc. In this work, we report the results of calcium phosphate nanoparticles obtained from calcined fish bones using laser ablation in de-ionized water. This technique offers some advantages: direct formation of nanoparticles in solutions, the absence of contamination, all particles are collected, easiness of preparation, low costs of processing, etc.

In previous works, we obtained calcium phosphate micro and nanoparticles from fish bones by laser ablation in ambient conditions [11] and laser-induced fracture [12]. In the present study, we report the production of β-TCP and HA nanoparticles from a natural source such as calcined fish bones.

## Experimental procedure

The powder used as starting material was obtained from fish bones according to the following procedure. The fish bones were boiled in water for 1 h and washed using a strong water jet to eliminate the fish meat. The washed fish bones were then dried and heated in air at 950°C for 12 h. The calcined samples were milled during 1 min. Pellets of the obtained product were prepared as precursor material to be ablated in de-ionized water by two different lasers operating at 1064 and 1075 nm wavelength, respectively. The first system used was a pulsed Nd:YAG laser delivering a maximum average power of 500 W. The laser beam was coupled to an optical fiber of 400 μm diameter and focused onto the upper surface of the target by means of 80 mm of focal length lens, where the spot diameter at normal incidence for a pulsed laser was about 0.14 mm. Other parameters were varied as follows: laser pulse width 1 to 3 ms, frequency 5 to 10 Hz, and pulse energy 2 to 8 J. The second laser system used was a monomode Ytterbium-doped fiber laser. This laser works in continuous wave mode delivering a maximum average power of 200 W. Its high beam quality allowed setting the irradiance range between 2 × 10^5 ^and 10^6 ^W/cm^2^. The laser beam was coupled to an optical fiber of 50 μm diameter using the same focusing system and processing setup than in the case of the Nd:YAG laser. Precursor material was characterized by means of X-ray diffraction (XRD) using a Siemens D-500 equipment and by X-ray fluorescence (XRF) taken by a Siemens SRS 3000 unit. TEM, selected area electron diffraction (SAED), and HRTEM images were taken on a JEOL-JEM 210 FEG transmission electron microscope equipped with a slow digital camera scan, using an accelerating voltage of 200 kV, to reveal their crystalline. The morphology as well as the composition is described by the scanning electron microscopy (SEM) using a JEOL-JSM-6700F

## Results

**Figure 1:**
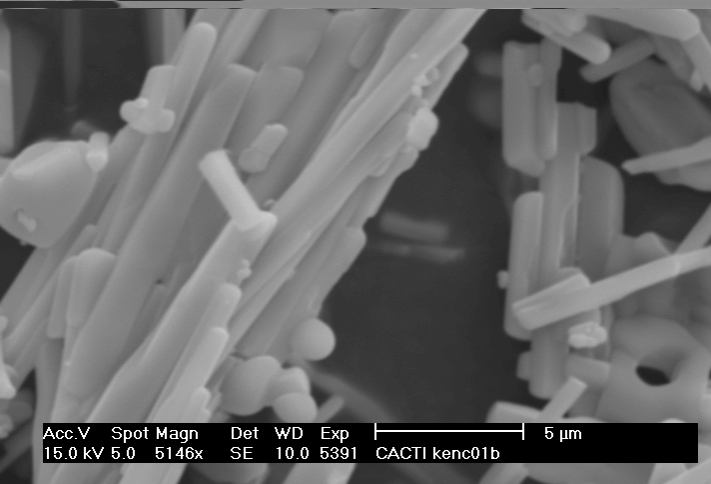
**SEM photograph of fish bones used as target showing the size and appearance of crystals**.

The starting material used as target submerged in de-ionized water consisted in pellets obtained from calcined fish bones. The calcined fish bones exhibited an appearance of rod-like with micrometric size as shown in Figure 1.

**Figure 2:**
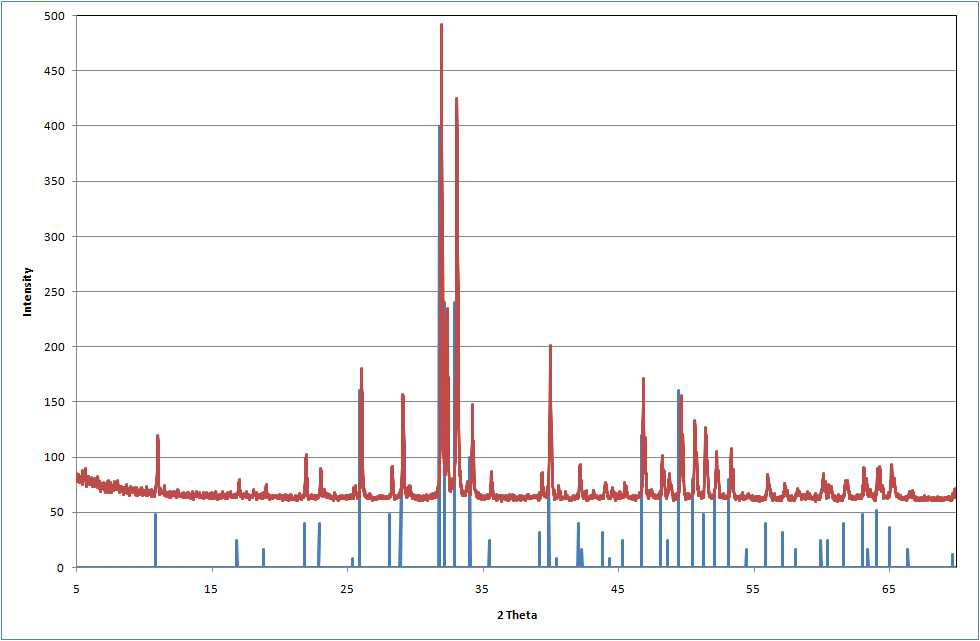
**XRD patterns of calcined fish bones compared with commercial stoichiometric HA (JCPDS 1993)**.

X-ray diffraction patterns of precursor materials compared with that of stoichiometric HA are shown in Figure 2. As can be seen, the biological material is composed of well crystallized HA. The composition detected by XRF revealed the presence of Ca and P as main elements in the samples with a Ca/P molar ratio of 1.68. Some minor elements were also detected, such as Mg, Na, Si, etc.

**Figure 3:**
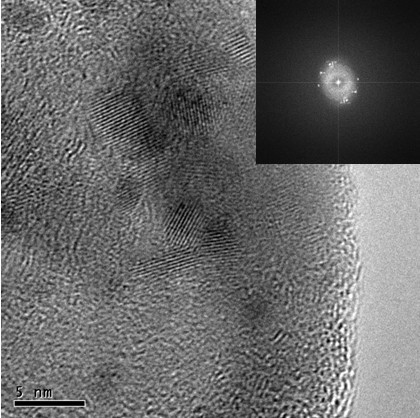
**HRTEM micrograph showing crystalline nanoparticles obtained from fish bones by laser ablation in water using pulsed laser and their corresponding fast Fourier transform (*inset*)**. Laser irradiance: 8 × 5 × 10^6 ^W/cm^2^.

The use of pulsed laser with 3 ms pulse width, 1.8 J, and 10 Hz of frequency (laser irradiance: 8 × 5 × 10^6 ^W/cm^2^) lead mainly to the formation of particles with rounded shape and nanometric size as can be seen from Figure 3. The HRTEM micrograph demonstrates that these particles are crystalline, showing the lattice fringes used to quantify the inter-planar spacing by means of the fast Fourier transform. The results of crystalline phases identified by inter-planar distances revealed that the obtained nanoparticles are mainly composed of HA and β-TCP, as listed in Table 1. The microanalysis performed on this kind of particles showed also the presence of trace elements, such as Mg and Si.

**Table 1:** The experimental inter-planar spacing of crystalline nanoparticles obtained from fish bones by pulsed laser ablation in water (laser irradiance: 8 × 5 × 10^6 ^W/cm^2^) compared to the correspondence to HA and β-TCP

Experimental (*d_hkl _*nm)	(*d_hkl _*nm) JCPDS_ICDD(1993)
0.238	0.230 (HA)
0.242	0.242 (β-TCP)
0.250	0.253 (HA)

**Figure 4:**
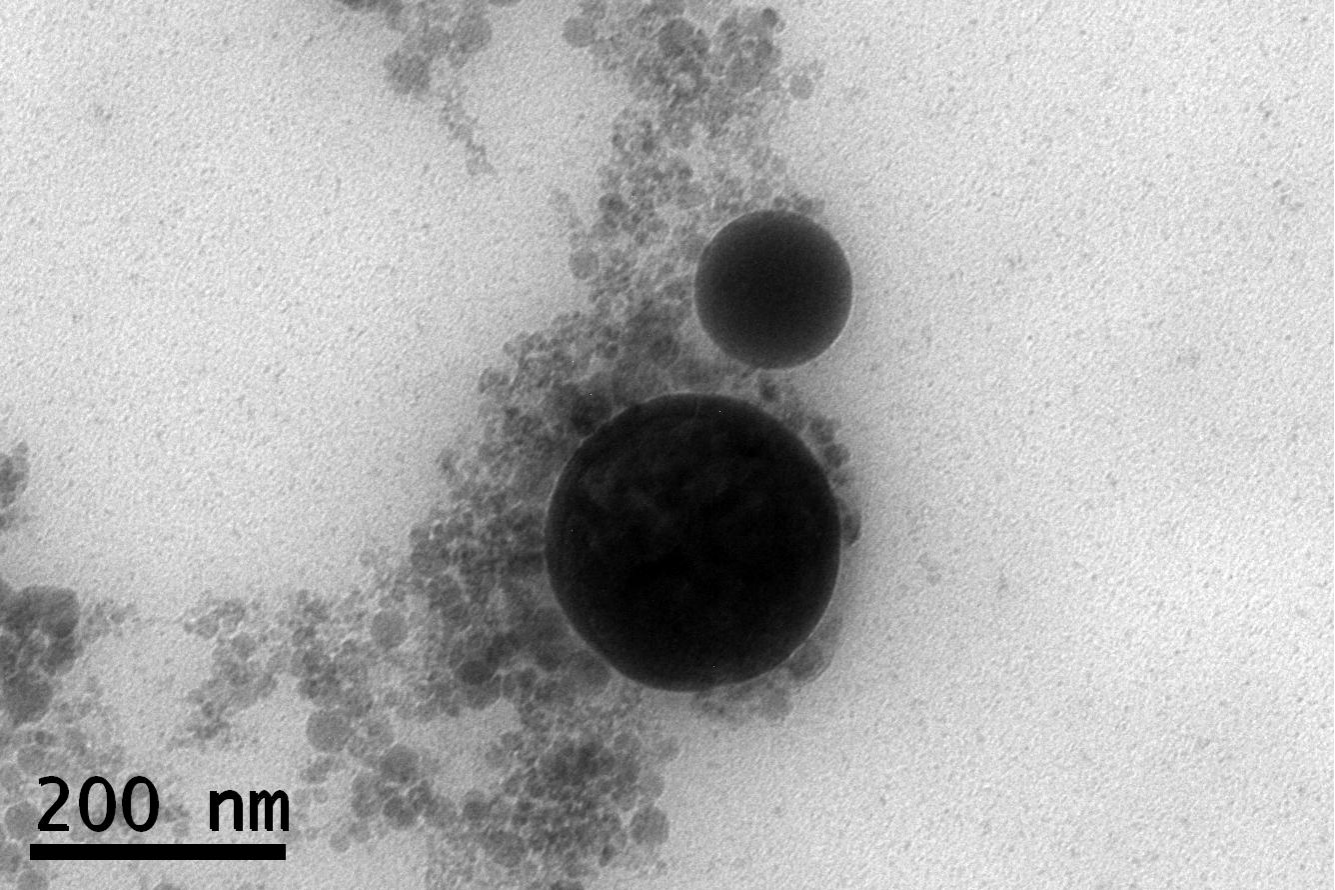
**HRTEM micrograph showing amorphous nanoparticles and submicronic particles obtained from fish bones by laser ablation in water using CW laser**. Laser irradiance: 6 × 10^5 ^W/cm^2^.

**Figure 5:**
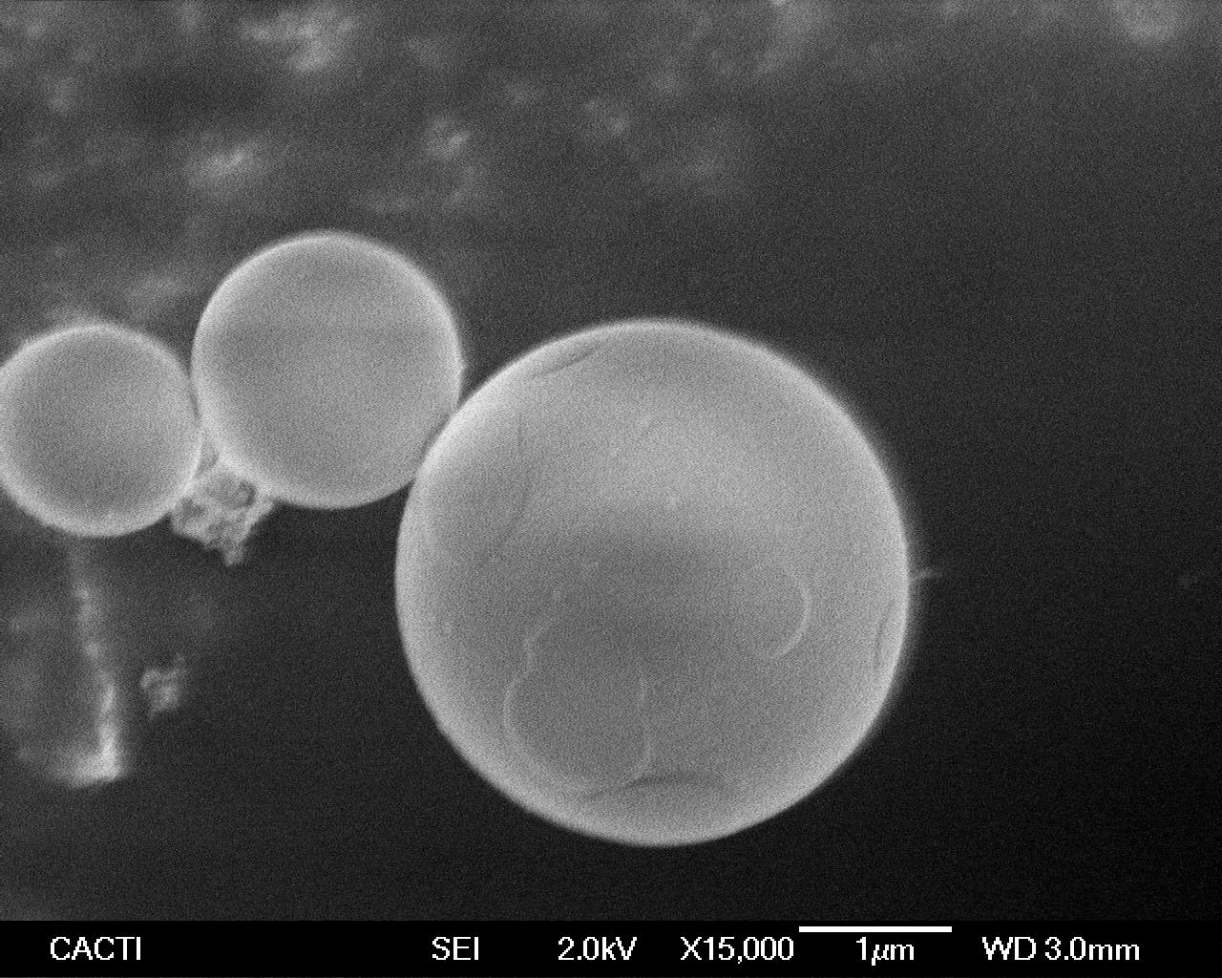
**SEM micrograph of micrometric particles obtained from fish bones by laser ablation in water using CW laser**. Laser irradiance: 6 × 10^5 ^W/cm^2^.

The use of continuous wave Yb:YAG fiber laser at irradiances around 6 × 10^5 ^W/cm^2 ^led to the formation of particles with spherical shape ranging from nanometric to micrometric size (Figures 4 and 5), but the predominance of the nanometric ones is evident from Figure 4. According to the results of the SAED performed on a group of this kind of particles they are amorphous. Nevertheless, the microanalysis performed on groups of these particles revealed the presence of the same elements of precursor material.

## Discussion

When a material surface is excited by laser irradiation, the photon energy is converted to heat due to photon-atom interaction, leading to a rapid temperature rise. As a result, a plume formed by high energetic species can be generated, where the amount of the mass removed and the energy of the laser are involved in a complex process, which depends on the laser parameters (pulse duration, energy, wavelength, etc.), the solid target properties, and the surrounding environment [13]. Due to the high energy density reached at the target surface, several changes may occur, such as vaporization, surface melting into a liquid with a moving solid-liquid interface, and for some materials thermal stress effects are important since they may cause the surface fracture of the solid [14]. All these mechanisms can contribute to the formation of particles, which can be obtained from condensation of evaporated material, from solidification of liquid droplets ejected by the recoil pressure induced by vaporization, as well as fragmented material from the target. According to TEM and SEM observations, there are differences among the particles obtained with the pulsed and the CW laser. However, the majority of obtained particles in both cases are spherical, which means they are probably formed by explosive ejection due to the high temperature reached at the zone interaction [15,16] or melting and rapid solidification. In thermal confinement regime, pulse duration is shorter than the time needed for heat dissipation in target (τ_p _≪ *t*_tc_). Under this condition, pulse duration is shorter than the time needed for bubbles formation and diffusion in the process of heterogeneous boiling [17,18]; therefore, the material can be overheated over the boiling temperature leading to explosive vaporization at low fluences or phase explosion at higher fluences [19,20]. On the other hand, stress confinement condition is fulfilled when the energy is deposited in the irradiated volume more rapidly than it can be dissipated through collective molecular motion according to τ_p _≪ *t*_sc_, which can lead to material fracture into more or less chunks [21]. As the use of CW laser is dominated by thermal regime, both conditions can be estimated in our work in the case of pulsed laser when thermal diffusivity () and the speed of sound (*v*_s _*= *1801 m/s) for spongy bone [22,23] are assumed valid for fish bones. Calculations for the used laser beam diameter *(*φ = 0.14 mm) confirm that thermal confinement condition is fulfilled for laser pulse durations in our experiments. The characteristic time *t*_ch _for heat dissipation in fish bones can be estimated according to  where *d *is the smallest dimension of the heated volume (beam diameter) and α is the thermal diffusivity, resulting in *t_tc _*= 18 ms, which is considerably longer than the used laser pulses. On the other hand, stress confinement characteristic can be estimated as  giving *t_sc _*= 83 ns, which is orders of magnitude shorter than the used pulse durations. This corroborates that thermal confinement is the only mechanism responsible for material explosive ejection and subsequent particles formation, which is consistent with the size as well as the spherical shape of the obtained particles. As the irradiance when using the pulsed laser is higher than when using the CW laser, the particles obtained in the latter conditions are amorphous, while the obtained in the former case are crystalline; other authors have obtained crystalline hydroxylapatite particles using pulsed laser ablation at higher irradiance [24,25].

Concerning the composition, crystalline particles obtained by the use of pulsed laser still preserve the composition of precursor material, although some of them undergo transformation phase from precursor HA to β-TCP promoted by longer pulse and high temperature. The effect of laser irradiation is expected to induce structural changes in material precursor constituted by HA due to the elevated temperature. Investigations in enamel irradiated with laser reported the formation of traces of α-TCP phase [26] when CO_2 _laser is used and the presence of traces of α-TCP and β-TCP when the source is Nd:YAG laser [27], which are in accordance with the obtained results. The amorphous particles obtained when using CW laser are calcium phosphate compounds, probably formed by melting and rapid solidification due the low irradiance delivered by the CW laser.

## Conclusions

In summary, we have been obtained HA and β-TCP nanoparticles by the use of laser ablation of targets from fish bones suspended in de-ionized water. The particles were obtained using pulsed as well as continuous wave laser. The use of the first one promotes the crystalline nanoparticles formation due to the high irradiance, while the latter one favors the formation of amorphous particles. The formation mechanism of particles can be attributed to explosive ejection.

## Competing interests

The authors declare that they have no competing interests.

## Authors' contributions

MB and JP conceived the work. MB and RC performed the experiments with the Nd:YAG laser. MB and AR performed the experiments with the fiber laser. Characterization of materials was carried out by FL. JP directed the work and wrote the draft paper. All authors contributed to the interpretation of results, discussion and read, corrected and approved the final manuscript.

## Abbreviations

CW: continuous wave; SAED: selected area electron diffraction; SEM: scanning electron microscopy; TEM: transmission electron microscopy; XRD: X-ray diffraction; XRF: X-ray fluorescence.

## References

[B1] Wang W Shi D Lian J Liu G Wang L Ewing RC Luminescent hydroxylapatite nanoparticles by surface functionalization Appl Phys Lett 2006 89 183106

[B2] Aronov D Rosenman G Karlov A Shashkin A Wettability patterning of hydroxyapatite nanobioceramics induced by surface potential modification Appl Phys Lett 2006 88 163906

[B3] Xu HHK Weir MD Sun L Takagi S Chow LC Effects of calcium phosphate nanoparticles on Ca-PO_4 _composite J Dent Res 2007 86 378 383 10.1177/154405910708600415 PMC2646419 17384036

[B4] Barinov SM Baschenko YV Krajewski A Application of ceramic composites as implants: result and problem Bioceramics and the human body 1992 UK: Elsevier Science Publishers 206 210

[B5] Koshino T Murase T Takagi T Saito T New bone formation around porous hydroxyapatite wedge implanted in opening wedge high tibial osteotomy in patients with osteoarthritis Biomaterials 2001 22 1579 1582 10.1016/s0142-9612(00)00318-5 11374457

[B6] Schneider OD Loher S Brunner TJ Uebersax L Simonet M Grass RN Merkle HP Stark WJ Cotton wool-like nanocomposite biomaterials prepared by electrospinning: in vitro bioactivity and osteogenic differentiation of human mesenchymal stem cells J Biomed Mater Res B Appl Biomater 2007 84B 350 362 10.1002/jbm.b.30878 17618506

[B7] Roohani-Esfahani SI Nouri-Khorasani S Lu Z Appleyard R Zreiqat H The influence hydroxyapatite nanoparticle shape and size on the properties of biphasic calcium phosphate scaffolds coated with hydroxyapatite-PCL composites Biomaterials 2010 31 5498 5509 10.1016/j.biomaterials.2010.03.058 20398935

[B8] Yubao L De Groot K De Wijn J Klein CPAT Meer SVD Morphology and composition of nanograde calcium phosphate needle-like crystals formed by simple hydrothermal treatment J Mater Sci Mater Med 1994 5 326 331

[B9] Yao J Tjandra W Chen YZ Tam KC Ma J Soh B Hydroxyapatite nanostructure material derived using cationic surfactant as a template J Mater Chem 2003 13 3053 3057

[B10] Liu J Li K Wang H Zhu M Xu H Yan H Self-assembly of hydroxyapatite nanostructures by microwave irradiation Nanotechnology 2005 16 82 87

[B11] Boutinguiza M Lusquiños F Comesaña R Riveiro A Quintero F Pou J Production of microscale particles from fish bone by gas flow assisted laser ablation Appl Surf Sci 2007 254 1264 1267

[B12] Boutinguiza M Lusquiños F Riveiro A Comesaña R Pou J Hydroxylapatite nanoparticles obtained by fiber laser-induced fracture Appl Surf Sci 2009 255 5382 5385

[B13] Park HK Haglund RF Laser ablation and desorption from calcite from ultraviolet to mid-infrared wavelengths Appl Phys A 1997 64 431 438

[B14] Chen Y Bulatov V Singer L Stricker J Schechter I Mapping and elemental fractionation of aerosols generated by laser-induced breakdown ablation Anal Bioanal Chem 2005 383 1090 1097 10.1007/s00216-005-0126-2 16283266

[B15] Kotaidis V Dahmen C Von Plessen G Excitation of nanoscale vapor bubbles at the surface of gold nanoparticles in water J Chem Phys 2006 124 184702 10.1063/1.2187476 16709126

[B16] Kotaidis V Plech A Cavitation dynamics on the nanoscale Appl Phys Lett 2005 87 213102

[B17] Miotello A Kelly R Laser-induced phase explosion: new physical problems when a condensed phase approaches the thermodynamic critical temperature Appl Phys A Mater Sci Process 1999 69A S67 S73

[B18] Kelly R Miotello A Contribution of vaporization and boiling to thermal-spike sputtering by ions or laser pulses Phys Rev E 1999 60 2616 2625 10.1103/physreve.60.2616 11970063

[B19] Zhigilei LV Garrison BJ Molecular dynamics simulation study of the fluence dependence of particle yield and plume composition in laser desorption and ablation of organic solids Appl Phys Lett 1999 74 1341 1343

[B20] Zhigilei LV Kodali PBS Garrison BJ On the threshold behavior in the laser ablation of organic solids Chem Phys Lett 1997 276 269 273

[B21] Paltauf G Dyer PE Photomechanical processes and effects in ablation Chem Rev 2003 103 487 518 10.1021/cr010436c 12580640

[B22] Peña G Calderón A Muñoz RA Stolik S Cruz A Sánchez F Microestructura y estudio de la difusión de calor en hueso y metales de uso biomédico Sup y Vacío 2000 11 70 73

[B23] Hatakeyama R Yoshizawa M Moriya T A method for the measurement of acoustic impedance and speed of sound in a small region of bone using a fused quartz rod as a transmission line Jpn J Appl Phys 2000 39 6449 6454

[B24] Mhin SW Ryu JH Kim KM Simple synthetic route for hydroxyapatite colloidal nanoparticles via a Nd:YAG laser ablation in liquid medium Appl Phys A 2009 96 435 440

[B25] Musaev OR Dusevich V Wieliecza DM Wrobel JM Kruger MB Nanoparticle fabrication of hydroxyapatite by laser ablation in water J Appl Phys 2008 104 084316 1 5

[B26] Nelson DGA Wefel Jongebloed WL Featherstone JDB Morphology, histology and crystallography of human dental enamel treated with pulsed low-energy infrared laser radiation Caries Res 1987 21 411 426 10.1159/000261047 3477323

[B27] Linc C Lee B Lin F Kork S Lan W Phase, Compositional and morphological changes of human dentin after Nd:YAG laser treatment J Endodont 2001 27 389 393 10.1097/00004770-200106000-00004 11487131

